# Progression from new methicillin-resistant *Staphylococcus aureus* colonisation to infection: an observational study in a hospital cohort

**DOI:** 10.1186/1471-2334-13-491

**Published:** 2013-10-22

**Authors:** Michelle ND Balm, Andrew A Lover, Sharon Salmon, Paul A Tambyah, Dale A Fisher

**Affiliations:** 1 Infection Control Team, National University Hospital, Singapore, Singapore; 2 Microbiology, Department of Laboratory Medicine, National University Hospital, Singapore, Singapore; 3 Infectious Diseases Programme, Saw Swee Hock School of Public Health, National University of Singapore, Singapore, Singapore; 4 Division of Infectious Diseases, University Medicine Cluster, National University Hospital, Singapore, Singapore; 5 Department of Medicine, Yong Loo Lin School of Medicine, National University of Singapore, Singapore, Singapore

**Keywords:** MRSA, Colonisation, Hospital-associated infection

## Abstract

**Background:**

Patients newly colonised with methicillin-resistant *Staphylococcus aureus* (MRSA) are at higher risk of clinical MRSA infection. At present, there are limited data on the duration or magnitude of this risk in a hospital population with a known time of MRSA acquisition.

**Methods:**

A retrospective cohort study of 909 adult patients known to have newly identified MRSA colonisation during admission to National University Hospital, Singapore between 1 July 2007 and 30 June 2011 was undertaken. Patients were excluded if they had history of previous MRSA colonisation or infection, or if they had been a hospital inpatient in the preceding 12 months. Data were collected on the development of MRSA infection requiring hospitalisation up to 30 June 2012.

**Results:**

Of 840 patients newly colonised with MRSA as identified on active surveillance and not clinical specimens, 546 were men (65.0%) and the median age was 65 years (range 18–103 years). Median follow up was 24 months (range 0 –64 months, 85.1% followed >6 months). Clinical infection occurred in 121 patients (14.4%) with median time to infection of 22 days (95% CI 14–31). Overall 71.9% (87/121) of infected patients developed infection within 60 days of the date MRSA colonisation was detected. However, 17/121 patients (14.0%) developed clinical infection more than six months after documented MRSA acquisition. The most common sites of clinical infection were skin and soft tissue (49/121, 40.5%, 95% CI 31.7-49.8), respiratory tract (37/121, 30.6%, 95% CI 22.5-39.6) and bone and joint infections (14/121, 11.6%, 95% CI 6.5-18.7). Thirteen patients (13/121, 10.7%, 95% CI 5.8-17.7) had bacteraemias, of which six (5.0% 95% CI 1.8-10.5) were primary and seven (5.7%, 95% CI 2.3-11.6) were secondary to infection at other sites. Crude mortality at 30 days and six months was higher in patients with MRSA infection than colonisation alone (aOR 5.49, 95% CI 2.75-10.95, p<0.001 and aOR 2.94, 95% CI 1.78-4.85, p<0.001 respectively).

**Conclusion:**

Risk of clinical infection is highest soon after MRSA acquisition. Prevention of MRSA acquisition in hospital will have significant impact on morbidity and mortality for patients.

## Background

Methicillin-resistant *Staphylococcus aureus* (MRSA) has become a major cause of hospital-associated infection since emerging in the 1960s. According to National Healthcare Safety Network data, in 2009–2010 MRSA accounted for 8.5% of all hospital-associated infections in the United States and was the most frequent multiresistant organism causing hospital-associated infection [1].

MRSA infection has been associated with many negative outcomes including higher hospital costs, longer hospital stays and higher mortality [2,3]. This has been shown with a variety of infection sites and a range of hospital contexts, including our own. In Singapore, patients with MRSA infection during admission were 10.2 times more likely to die during hospitalisation, had 4.6 times longer hospital stays and had hospitalisation costs 4.0 times higher than matched uninfected controls [4].

Infections with MRSA following initial colonisation are known to be due to the same strain in most patients [5]. Colonisation with MRSA is a major risk factor for subsequent MRSA infection [6-8]. This is well-established for infection occurring in the same admission as MRSA detection, and there is mounting evidence that the risk of MRSA infection may persist for longer periods in some colonised patients [9-13].

However, most studies have evaluated patients who have MRSA without separately analysing incident and prevalent MRSA carriers. Furthermore, few studies have followed patients for more than eighteen months. To date there are limited data on the impact of newly acquired MRSA in a tertiary hospital setting. We sought to investigate the risk of progression to infection over a five year period in a cohort of patients who acquired MRSA during an admission at our hospital.

## Methods

### Setting

National University Hospital (NUH) is a 1000 bed tertiary hospital in Singapore. An MRSA control bundle which included active surveillance cultures (ASC) at admission and discharge was introduced in Intensive Care Units (ICUs) in 2007. The bundle was progressively expanded throughout the hospital over subsequent years such that by 2011, universal ASC on ward admission and discharge were routine on all adult inpatient wards except psychiatry and obstetrics. Patients transferring between wards underwent ASC upon transfer (e.g. from ICU to general ward). Patients colonised with MRSA were not routinely decolonised during this study.

### Design

We conducted a retrospective cohort study to evaluate the risk of progression to infection in adult patients who had newly acquired MRSA during an inpatient admission to NUH between 1 July 2007 and 30 June 2011. Patients were included if they had negative ASC on admission but subsequent positive ASC during the same admission. Patients were excluded if they had been admitted to any healthcare facility within the preceding 12 months or if they had a history of previous MRSA colonisation or infection. Analysis was not performed on patients that developed MRSA infection during their index admission if the clinical isolate grew MRSA prior to the screen swab. That is, patients who appeared to develop infection without evidence of prior colonisation were not included. Only Singapore citizens or permanent residents were included in the study to avoid likely loss to follow up. Patient records were reviewed until death or 30 June 2012 to determine if clinical MRSA infection developed. In this way, we aimed to investigate the risk of progression to infection in a group of patients in whom we could reasonably attribute MRSA acquisition to a specific admission at our hospital.

ASC were taken using one swab from both nares, and one from both axillae and groin on admission. Swabs were processed as a pooled sample and inoculated on chromogenic media (MRSA Select, Bio-Rad Laboratories, Marnes-la-Coquette, France) with aerobic incubation for up to 48 hours. Clinical specimens were taken at the discretion of the primary team according to routine care and processed using existing laboratory protocols based on the type of sample. Prior to 2010, MRSA identification was confirmed using Vitek2 instrument (bioMérieux, Marcy L’Etoile, France), and from 2010 onwards, by matrix assisted laser desorption ionisation-time of flight (MALDI-TOF, Bruker Daltoniks GmbH, Bremen, Germany).

Data were collected retrospectively on demographics (age, gender, ethnicity) and pre-existing co-morbidities (diabetes, active malignancy, non-cancer immunosuppression, chronic haemodialysis). Data on healthcare utilisation during the admission in which MRSA was acquired were also collected. This was limited to information stored electronically but included ICU admission, surgery within 30 days of MRSA acquisition, and intravascular catheter placement. Co-morbidities were classified using International Classification of Diseases, Ninth Revision codes from discharge summary records. Patients developing MRSA clinical infection were further evaluated as to the primary site of the infection according to NHSN definitions [14], need for admission and number of days between first positive MRSA ASC and first positive culture from a clinical site (i.e. not a surveillance culture). Outcome data on re-admission due to MRSA infection at any public hospital in Singapore and for mortality at one and six months were reviewed.

The study was approved by the Domain Specific Review Board for the National Healthcare Group institutions in Singapore (NHG DSRB 2012/00513).

### Statistical analysis

Bivariate analysis was first performed to identify variables that showed significance at the 0.20 level with a binary outcome, with the exceptions of age, gender and ethnicity which were forced into the model to address the possibility of residual confounding. To verify an association between categorical variables, the χ-square test was employed. Akaike and Bayesian Information Criterion (AIC/BIC) were used as primary drivers for model building, and the model fit was checked with the Hosmer-Lemeshow goodness-of-fit test (by deciles). Additionally, receiver-operating curve (ROC) plots and sensitivity/specificity tables were utilised to assess the practical utility of the individual logistic models. Kaplan-Meier analysis was performed to identify the unadjusted time-to-event with associated confidence intervals within the entire cohort. Data analysis was performed using Stata 12.1 (College Station, Texas); all tests were two-tailed.

## Results

**Figure 1:**
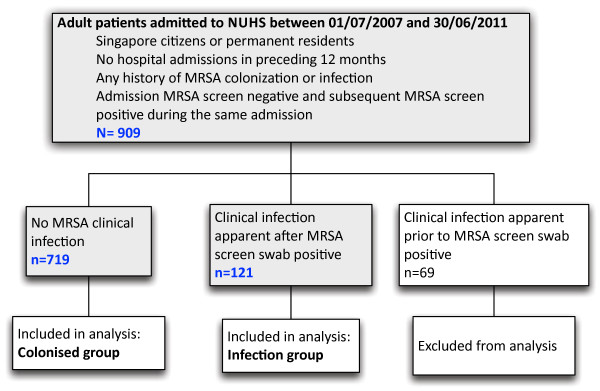
**Study design.** Study design and distribution of study population.

From 1 July 2007 to 30 June 2011, 228968 patients were admitted to NUH. Their average length of stay was 5.6 days. During this time, we identified 909 patients on routine screening who were entry ASC negative but acquired MRSA colonisation during their hospitalisation. Of these, 69 patients also had MRSA isolated from clinical sites before being identified as MRSA colonised through ASC; these patients were not further analysed (Figure 1). Of the remaining 840 patients, 546 (65.0%) were men and the median age was 65 years (range 18–103 years). Ethnic representation approximated the national distribution with 68% Chinese, 18% Malay, 9% Indian and 5% other (Table 1). The median time between admission (negative ASC) and known colonisation (first detected positive ASC) was 11 days. Patient records were reviewed until death or 30 June 2012; the median duration of this review was 24 months and 85.1% (715/840) were followed for >6 months.

**Table 1:** Demographic and clinical characteristics of colonised (n=716) and infected patients (n=121)

	**Colonised patients (%)**		**Infected patients (%)**		**Total (%)**	
**Age (years)**						
**≤ 39**	83	(11.5%)	7	(5.8%)	90	(10.7%)
**40-59**	209	(29.1%)	30	(24.8%)	239	(28.5%)
**60-79**	298	(41.4%)	65	(53.7%)	363	(43.2%)
**80 +**	129	(17.9%)	19	(15.7%)	148	(17.6%)
**Gender**						
**Male**	460	(64.0%)	86	(71.1%)	546	(65.0%)
**Female**	259	(36.0%)	35	(28.9%)	294	(35.0%)
**Ethnicity**						
**Chinese**	485	(67.5%)	85	(70.2%)	570	(67.9%)
**Malay**	131	(18.2%)	20	(16.5%)	151	(18.0%)
**Indian**	65	(9.0%)	11	(9.1%)	76	(9.0%)
**Other**	38	(5.3%)	5	(4.1%)	43	(5.1%)
**Co-morbidities**						
**Diabetes**	233	(32.4%)	45	(37.2%)	278	(33.1%)
**Active malignancy**	66	(9.2%)	18	(14.9%)	84	(10.0%)
**Haemodialysis**	246	(34.2%)	49	(40.5%)	295	(35.1%)
**Non-cancer immunosuppression**	91	(12.7%)	2	(1.7%)	93	(11.1%)
**Exposures**						
**Surgery within 30 days**	350	(48.7%)	71	(58.7%)	421	(50.1%)
**ICU admission**	325	(45.2%)	67	(55.4%)	392	(46.7%)
**Central line placement**	237	(33.0%)	50	(41.3%)	287	(34.2%)
**Outcome**						
**Mortality at 30 days**	33	(4.6%)	20	(16.5%)	53	(6.3%)
**Mortality at 6 months**	90	(12.5%)	35	(28.9%)	125	(14.9%)

**Figure 2:**
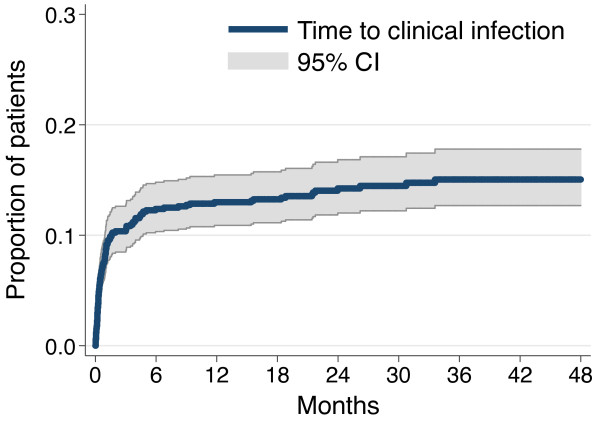
**Kaplan-Meier estimates of clinical MRSA infection from time of colonization (months) (n=840).** Kaplan-Meier estimate showing proportion of patients with clinical MRSA infection shown at six-monthly time intervals.

Clinical MRSA infection developed in 121 of 840 patients (14.4%) within the follow up period. The median time to infection was 22 days from the time of positive ASC (95% CI 14–31). The majority of patients developing clinical infection did so soon after acquisition, with 42.1% (51/121, 95% CI 33.2-51.5) presenting with infection within 14 days of MRSA acquisition (Figure 2). This increased to 57.8% (70/121, 95% CI 49.4-67.6) by 30 days and 71.9% (87/121, 95% CI 63.0-79.7) at two months. Eighty-two patients (82/121, 67.8%, 95% CI 58.7-76.0) developed MRSA infection after discharge and had the infection detected at an outpatient clinic visit or at a subsequent admission. This included a minority of patients with onset of infection more than six months after documented MRSA acquisition (17/121, 14.0%, 95% CI 8.4-21.5). Alternatively from a patient perspective: if MRSA infection had not occurred within 60 days of colonisation then we observed the future risk of MRSA infection to be just 4% (34/840, 95% CI 2.8-5.6).

The most common sites of clinical infection were skin and soft tissue (49/121, 40.5%, 95% CI 31.7-49.8), respiratory tract (37/121, 30.6%, 95% 22.5-39.6) and bone and joint infections (14/121, 11.6%, 95% CI 6.5-18.7). Thirteen patients (13/121, 10.7%, 95% CI 5.8-17.7) had bacteraemias, of which six (5.0% 95% CI 1.8-10.5) were primary and seven (5.7%, 95% CI 2.3-11.6) were secondary to infection at other sites.

Surgery within 30 days (OR 1.50, 95% CI 1.01-2.21, p=0.043) as well as admission to ICU during the index admission (OR 1.50, 95% CI 1.02 – 2.22, p=0.039) were associated with MRSA infection (Table 2). Patients who were immunosuppressed (non-malignancy related immunosuppression) appeared to have a lower risk of developing infection if colonised (OR 0.11, CI 0.028-0.478, p=0.002). No demographic or medical exposures apart from age were associated with development of clinical infection on multivariate logistic regression analysis.

**Table 2:** Risk factors for clinical infection (n=840) (Outcome defined as MRSA-positive clinical infection)

	**Crude odds ratio (95% C.I.)**	**Adjusted odds ratio (95% C.I.)**	**p value**
**Age (years)**			
≤ 39	1.0 (ref)	1.0 (ref)	-
40-59	1.70 (0.72 – 4.03)	1.85 (0.78 – 4.40)	0.163
60-79	**2.59 (1.14 - 5.85)**	**2.90 (1.27 – 6.61)**	**0.012**
80 +	1.75 (0.70 – 4.33)	2.15 (0.85 – 5.45)	0.106
**Gender**			
Male	1.0 (ref)	1.0 (ref)	-
Female	0.73 (0.47 – 1.10)	0.67 (0.43 – 1.03)	0.066
**Ethnicity**			
Chinese	1.0 (ref)	1.0 (ref)	-
Malay	0.87 (0.52 - 1.47)	0.93 (0.54 -1.60)	0.799
Indian	0.97 (0.49 - 1.90)	1.01 (0.51 – 2.04)	0.959
Other	0.75 (0.29 - 1.96)	0.86 (0.32 - 2.31)	0.772
**Co-morbidities**			
Immunosuppression	**0.11 (0.03 - 0.48)**	**0.11 (0.03 - 0.44)**	**0.002**

Bolded entries are significant at p < 0.05.

Clinical infection seemed more likely to develop in those aged 60–79 years, and was more common in males although these trends did not reach statistical significance. In this cohort of patients with known time of MRSA acquisition, we did not identify any comorbid conditions or exposures during their admission that were associated with increased risk of developing subsequent MRSA infection. However, MRSA infection was strongly associated with mortality (Table 3). Of the 840 patients studied, 125 died within six months of MRSA acquisition. In patients developing MRSA infection after being colonised, 16.5% (20/121) died either during admission or within 30 days of discharge from the admission in which they acquired MRSA. An additional 15 patients who developed MRSA infection (15/121, 12.4%, 95% CI 10.4-24.4) died within six months of MRSA acquisition. MRSA infection increased the risk of death at 30 days over fivefold compared to patients who were only colonised (aOR 5.49, 95% CI 2.75-10.95, p<0.001). Risk of death remained elevated at six months (aOR 2.94, 95% CI 1.78-4.85, p<0.001). Of patients surviving to discharge, those who developed MRSA infection were less likely to be discharged to their own home (p=0.04) and 32/121 patients (26.4%) required multiple admissions for complications of MRSA infections.

**Table 3:** Risk factors for mortality (n=840) (Outcome defined as all-cause mortality)

	**Mortality at 30 days**		**Mortality at six months**	
	**Adjusted odds ratio (95% C.I.)**	**p value**	**Adjusted odds ratio (95% C.I.)**	**p value**
**Infection group**				
Clinical MRSA	**5.49 (2.75- 10.95)**	**< 0.001**	**2.94 (1.78 – 4.85)**	**< 0.001**
**Age (years)**				
≤ 39	1.0 (ref)	-	1.0 (ref)	-
40-59	0.61 (0.11 – 3.52)	0.584	2.28 (0.50 – 10.34)	0.285
60-79	1.91 (0.40 – 9.06)	0.417	**5.17 (1.21 – 22.02)**	**0.026**
80 +	2.53 (0.50 – 12.74)	0.260	**16.45 (3.78 – 71.50)**	**< 0.001**
**Gender**				
Male	1.0 (ref)	-	1.0 (ref)	-
Female	**1.92 (1.03 – 3.60)**	**0.040**	0.98 (0.63 – 1.51)	0.919
**Ethnicity**				
Chinese	1.0 (ref)	-	1.0 (ref)	-
Malay	0.39 (0.13 – 1.18)	0.096	0.55 (0.27 – 1.11)	0.096
Indian	1.03 (0.32 – 3.30)	0.964	0.97 (0.46 to 2.08)	0.936
Other	0.46 (0.05 – 4.04)	0.479	0.42 (0.09 – 1.85)	0.251
**Co-morbidities**				
Malignancy	**2.29 (1.05 – 5.03)**	**0.038**	**2.32 (1.33 – 4.04)**	**0.003**
Immunosuppression	**2.98 (1.30 – 6.80)**	**0.010**	NS	NS
**Inpatient risk factors**				
Surgery ≤ 30 days	**0.35 (0.17 – 0.69)**	**0.003**	**0.56 (0.35 -0.90)**	**0.016**
ICU	2.50 (0.98 - 6.39)	0.055	NS	NS
CVC	**3.82 (1.60 – 9.12)**	**0.003**	**2.40 (1.51 – 3.83)**	**< 0.001**

Bolded entries are significant at p < 0.05.

## Discussion

This study shows that the highest risk for MRSA infection occurs shortly after patients become colonised. However, a significant proportion of these patients will present with their infection in a different admission to that in which they became colonised. Furthermore, there is a strong association between developing MRSA infection and death within six months. Our study is novel in that it evaluates a large cohort of patients with an identified likely time of MRSA acquisition. In this group, 14.4% progressed to infection with MRSA. Other studies, most of which did not distinguish between incident and prevalent carriers, have estimated this risk between 8.5% and 33% [6,7,10-13]. The risk of infection was much higher in the period around the time of the index admission shortly after the initial acquisition. Over 40% of those developing clinical infections did so within two weeks of first evidence of MRSA acquisition, and 72% presented within 60 days of first evidence of MRSA acquisition. Risk of infection appears highest in the first few months following MRSA acquisition. This situation may be similar to colonisation by *S. pneumoniae* and *N. meningitidis* where an interplay between host immunological factors and bacterial factors results in invasive disease occurring shortly after colonisation with a new bacterial strain, while some individuals remain colonised for years without infection [15,16]. In addition, healthcare associated risk factors such as the use of devices resulting in breach of normal host defences are likely to contribute to the development of infection. In our cohort recent surgery, ICU admission and central line placement were all associated with higher infection rates on bivariate analysis; however, these were not significant independent risk factors on multivariate analysis possibly due to the relatively small number of actual infections documented. Similar associations with MRSA infection have been found in other studies [7,12,17].

MRSA infections represent a considerable burden on the health care system. In our study, 67.8% (82/121) of infections presented in different admissions to the index admission in which the acquisition occurred, and 26.4% (32/121) of patients required multiple admissions for features or complications of MRSA infection. Other studies have recently highlighted the ongoing risk of infection following detection of MRSA colonisation [12,13]. In a large retrospective cohort of patients with newly detected MRSA colonisation or infection in the United States, 33% developed infections over the subsequent year and the majority of infections occurred after patients were discharged from hospital [13]. This is an important consideration with the tendency for shorter hospital stays as it places more reliance on post-discharge surveillance with adequate outpatient follow up of patients to detect and manage potential complications. Furthermore, 30 day all-cause mortality was over fivefold higher in MRSA colonised patients who developed clinical infections compared with those who did not, with significantly elevated risk of mortality persisting at six months also. Other studies have found a similar association between MRSA infection and mortality [10,13].

Prevention of MRSA acquisition is probably important in reducing subsequent development of MRSA infection and associated morbidity and mortality. Screening of patients to identify inpatient acquisition of MRSA colonisation is important in many settings, particularly if it is linked to infection prevention interventions that may reduce subsequent MRSA infection in these individuals. Surveillance programmes may be expensive in terms of resource use and financial costs, but can be cost effective in the long term [4,18,19]. Decolonisation of newly identified carriers, particularly in higher risk groups such as those with invasive devices or admitted to ICU, may be a useful addition to present standard strategies [19,20].

Strengths of this study include the large cohort of patients identified as acquiring MRSA during admission to a tertiary hospital. Excluding patients with any past history of MRSA and indeed any admission in the preceding 12 months strengthens the likelihood that the identification of acquisition was accurate. Patients were followed for up to five years (median 24 months), allowing evaluation of the longer-term risks of MRSA acquisition.

This is a single centre, retrospective study using electronic data collection methods. Frequency of ACS was limited to that performed routinely according to hospital policy. This poses some limitations on the study. We included patients who were likely to remain in the study region for the follow up period and captured data by search of shared electronic records regarding encounters at any public healthcare facility in Singapore, but we cannot search for admission to private hospitals or visits to community doctors. However, the referral patterns in Singapore with its small number of hospitals means that the number lost would not be large. We were also only able to capture data that was systematically collected on the electronic record. This limited some information regarding exposures including prior antibiotic use, residence in nursing homes prior to admission, use of nasogastric tubes or indwelling urinary catheters or presence of pressure ulcers and chronic wounds.

ASC were performed routinely on admission and discharge from wards by swabbing nares, axilla and groin and using direct culture on chromogenic media. Estimated sensitivity of direct culture on chromogenic media is approximately 84% when compared to PCR detection, and would also be dependent on sampling technique [21]. This could have resulted in some false negative results from patients who were prevalent carriers but swab negative on admission. Performing ASC weekly during admission or screening more sites (e.g. throat) may have provided more accuracy but also increase costs and been difficult to implement. Using time that MRSA colonisation was first detected as a proxy for actual MRSA acquisition may mask a time of undetected carriage however, we believe that for the majority of patients this was of very short duration and will not significantly affect the analysis. It is not routine practice at our hospital to perform ASC on outpatients, thus we could not follow patients to determine whether the MRSA colonisation was persistent. In addition, we could not control for other MRSA exposures after discharge from hospital. These may be important in patients with longer periods between detected MRSA colonisation and subsequent infection. Molecular typing on paired isolates representing colonisation and infection in the same patient would be useful to confirm the link between colonisation and infection. The local MRSA epidemiology in Singapore is virtually limited to two Multilocus Sequence Types (ST239 and ST22) [22]. Typing methodologies with greater discriminatory power such as Pulsed Field Gel Electrophoresis or Whole Genome Sequencing would therefore be required; however, only the first MRSA isolate from each patient was routinely stored by our hospital laboratory so pairs were not available for typing.

## Conclusion

In conclusion, our study shows that approximately 15% of patients who acquired MRSA developed a subsequent MRSA infection. Median time to infection was 22 days and the risk of infection in those colonised was highest in the peri-hospitalisation period. Patients developing MRSA infection were at risk of infection-related re-admission and had higher crude mortality rates than those patients without MRSA infection. These results confirm that prevention of MRSA acquisition in hospitals should be an important goal of programmes to reduce MRSA infections. At the same time, targeting interventions such as decolonisation therapy and enhanced efforts to prevent device-associated infection to reduce development of infection in patients who newly acquire MRSA colonisation may also have a key role. Since most infections occur soon after colonisation, useful further work would include the study of immune mechanisms that may offer partial longer term protection including potentially prophylactic or therapeutic vaccines.

## Abbreviations

ASC: Active surveillance culture; ICU: Intensive care unit; MRSA: Methicillin-resistant *Staphylococcus aureus*; NHSN: National Healthcare Safety Network; NUH: National University Hospital; PCR: Polymerase chain reaction.

## Competing interests

All authors declare they have no conflicts of interest.

## Authors’ contributions

MB participated in study design, performed the data extraction, data analysis and drafted the initial manuscript. AL performed the statistical analysis and provided critical appraisal of the manuscript. SS contributed to critical appraisal of the manuscript. PT participated in study design and critical appraisal of the manuscript. DF conceived the idea for the study, participated in its design and provided critical appraisal of the manuscript. All authors read and approved the final manuscript.

## Pre-publication history

The pre-publication history for this paper can be accessed here:

http://www.biomedcentral.com/1471-2334/13/491/prepub

## References

[B1] Sievert DM Ricks P Edwards JR Schneider A Patel J Srinivasan A Kallen A Limbago B Fridkin S Antimicrobial-resistant pathogens associated with healthcare-associated infections: summary of data reported to the National Healthcare Safety Network at the Centers for Disease Control and Prevention, 2009–2010 Infect Control Hosp Epidemiol 2013 13 1 14 23221186 10.1086/668770

[B2] Cosgrove SE Qi Y Kaye KS Harbarth S Karchmer AW Carmeli Y The impact of methicillin resistance in *Staphylococcus aureus* bacteremia on patient outcomes: mortality, length of stay, and hospital charges Infect Control Hosp Epidemiol 2005 13 166 174 15756888 10.1086/502522

[B3] Shorr AF Epidemiology and economic impact of meticillin-resistant *Staphylococcus aureus*: review and analysis of the literature Pharmacoeconomics 2007 13 751 768 17803334 10.2165/00019053-200725090-00004

[B4] Pada SK Ding Y Ling ML Hsu LY Earnest A Lee TE Yong HC Jureen R Fisher D Economic and clinical impact of nosocomial meticillin-resistant *Staphylococcus aureus* infections in Singapore: a matched case–control study J Hosp Infect 2011 13 36 40 21269733 10.1016/j.jhin.2010.10.016

[B5] Huang SS Diekema DJ Warren DK Zuccotti G Winokur PL Tendolkar S Boyken L Datta R Jones RM Ward MA Aubrey T Onderdonk AB Garcia C Platt R Strain-relatedness of methicillin-resistant *Staphylococcus aureus* isolates recovered from patients with repeated infection Clin Infect Dis 2008 13 1241 1247 18444862 10.1086/529381 PMC2723744

[B6] Davis KA Stewart JJ Crouch HK Florez CE Hospenthal DR Methicillin-resistant *Staphylococcus aureus* (MRSA) nares colonization at hospital admission and its effect on subsequent MRSA infection Clin Infect Dis 2004 13 776 782 15472807 10.1086/422997

[B7] Coello R Glynn JR Gaspar C Picazo JJ Fereres J Risk factors for developing clinical infection with methicillin-resistant *Staphylococcus aureus* (MRSA) amongst hospital patients initially only colonized with MRSA J Hosp Infect 1997 13 39 46 9321727 10.1016/s0195-6701(97)90071-2

[B8] Pujol M Pena C Palleres R Ayats J Ariza J Gudiol F Risk factors for nosocomial bacteremia due to methicillin-resistant *Staphylococcus aureus* Eur J Clin Microbiol Infect Dis 1994 13 96 102 8168571 10.1007/BF02026134

[B9] Huang SS Platt R Risk of methicillin-resistant *Staphylococcus aureus* infection after previous infection or colonization Clin Infect Dis 2003 13 281 285 12539068 10.1086/345955

[B10] Datta R Huang SS Risk of infection and death due to methicillin-resistant *Staphylococcus aureus* in long-term carriers Clin Infect Dis 2008 13 176 181 18532892 10.1086/589241 PMC2649874

[B11] Ramarathnam V De Marco B Ortegon A Kemp D Luby J Sreeramoju P Risk factors for development of methicillin-resistant *Staphylococcus aureus* infection among colonized patients Am J Infect Control 2013 doi:10.1016/j.ajic.0032012.08.005. in press 10.1016/j.ajic.2012.08.005 23290578

[B12] Quezada Joaquin NM Diekema DJ Perencevich EN Bailey G Winokur PL Schweizer ML Long-term risk for readmission, methicillin-resistant *Staphylococcus aureus* (MRSA) infection and death among MRSA-colonized veterans Antimicrob Agents Chemother 2013 13 1169 1172 23254427 10.1128/AAC.01968-12 PMC3591925

[B13] Huang SS Hinrichsen VL Datta R Spurchise L Miroshnik I Nelson K Platt R Methicillin-resistant *Staphylococcus aureus* infection and hospitalization in high risk patients in the year following infection PLoS One 2011 13 24340 doi:10.1371/journal.pone.0024340 10.1371/journal.pone.0024340 PMC3174953 21949707

[B14] Horan TC Andrus M Dudeck MA CDC/NHSN surveillance definition of health care-associated infection and criteria for specific types of infections in the acute care setting Am J Infect Control 2008 13 309 332 18538699 10.1016/j.ajic.2008.03.002

[B15] Bogaert D De Groot R Hermans PWM *Streptococcus pneumoniae* colonisation: the key to pneumococcal disease Lancet Infect Dis 2004 13 144 154 14998500 10.1016/S1473-3099(04)00938-7

[B16] Yazdankhah SP Caugant DA *Neisseria meningitidis:* an overview of the carriage state J Med Microbiol 2004 13 821 832 15314188 10.1099/jmm.0.45529-0

[B17] Fukata Y Cunningham CA Harris PL Wagener MM Muder RR Identifying the risk factors for hospital-acquired methicillin-resistant *Staphylococcus aureus* among patients colonized with MRSA on admission Infect Control Hosp Epidemiol 2012 13 1219 1225 10.1086/668420 23143359

[B18] Loveday HP Pellowe CM Jones SRLJ Pratt RJ A systematic review of the evidence for interventions for the prevention and control of meticillin-resistant *Staphylococcus aureus* (1996–2004): report to the Joint MRSA Working Party (Subgroup A) J Hosp Infect 2006 13 S45 S70 16616800 10.1016/j.jhin.2006.01.002

[B19] Robotham JV Graves N Cookson BD Barnett AG Wilson JA Edgeworth JD Batra R Cuthbertson BH Cooper BS Screening, isolation, and decolonisation strategies in the control of meticillin resistant *Staphylococcus aureus* in intensive care units: cost effectiveness evaluation Br Med J 2011 13 d5694 doi:10.1136/bmj.d5694 21980062 10.1136/bmj.d5694 PMC3188660

[B20] Simor AE Staphylococcal decolonisation: an effective strategy for prevention of infection? Lancet Infect Dis 2011 13 952 962 22115070 10.1016/S1473-3099(11)70281-X

[B21] Wolk DM Marx JL Dominguez L Driscoll D Schifman RB Comparison of MRSASelect agar, CHROMagar Methicillin-resistant *Staphylococcus aureus* (MRSA) medium and Xpert MRSA PCR for detection of MRSA in nares: Diagnostic accuracy for surveillance samples with various bacterial densities J Clin Microbiol 2009 13 3933 3936 19828738 10.1128/JCM.00601-09 PMC2786641

[B22] Teo J Tan TY Hon PY Lee W Koh TH Krishnan P Hsu LY ST22 and ST239 MRSA duopoly in Singaporean hospitals: 2006–2010 Epidemiol Infect 2013 13 153 157 22394568 10.1017/S0950268812000337 PMC9152064

